# Interplay of grounding-line dynamics and sub-shelf melting during retreat of the Bjørnøyrenna Ice Stream

**DOI:** 10.1038/s41598-018-25664-6

**Published:** 2018-05-08

**Authors:** Michele Petrini, Florence Colleoni, Nina Kirchner, Anna L. C. Hughes, Angelo Camerlenghi, Michele Rebesco, Renata G. Lucchi, Emanuele Forte, Renato R. Colucci, Riko Noormets

**Affiliations:** 1 OGS (Istituto Nazionale di Oceanografia e Geofisica Sperimentale), Borgo Grotta Gigante 42/c, 34010 Sgonico, TS Italy; 2 CMCC (Centro Euro-Mediterraneo sui Cambiamenti Climatici), via Franceschini 31, 40128 Bologna, BO Italy; 3 Bolin Centre for Climate Research, Stockholm University, SE-106 91 Stockholm, Sweden; 4 Department of Earth Science, University of Bergen and Bjerknes Centre for Climate Research, N-5020 Bergen, Norway; 5 Dipartimento di Matematica e Geoscience, Università di Trieste, Piazzale Europa 1, 34127 Trieste, TS Italy; 6 ISMAR (Istituto di Scienze Marine), Trieste, Italy; 7 The University Centre in Svalbard (UNIS), P.O. Box 156 Northern-9171, Longyearbyen, Norway; 8 Present Address: Department of Geoscience and Remote Sensing, Delft University of Technology (TUDelft), Delft, Netherlands

## Abstract

The Barents Sea Ice Sheet was a marine-based ice sheet, i.e., it rested on the Barents Sea floor during the Last Glacial Maximum (21 ky BP). The Bjørnøyrenna Ice Stream was the largest ice stream draining the Barents Sea Ice Sheet and is regarded as an analogue for contemporary ice streams in West Antarctica. Here, the retreat of the Bjørnøyrenna Ice Stream is simulated by means of two numerical ice sheet models and results assessed against geological data. We investigate the sensitivity of the ice stream to changes in ocean temperature and the impact of grounding-line physics on ice stream retreat. Our results suggest that the role played by sub-shelf melting depends on how the grounding-line physics is represented in the models. When an analytic constraint on the ice flux across the grounding line is applied, the retreat of Bjørnøyrenna Ice Stream is primarily driven by internal ice dynamics rather than by oceanic forcing. This suggests that implementations of grounding-line physics need to be carefully assessed when evaluating and predicting the response of contemporary marine-based ice sheets and individual ice streams to ongoing and future ocean warming.

## Introduction

**Figure 1:**
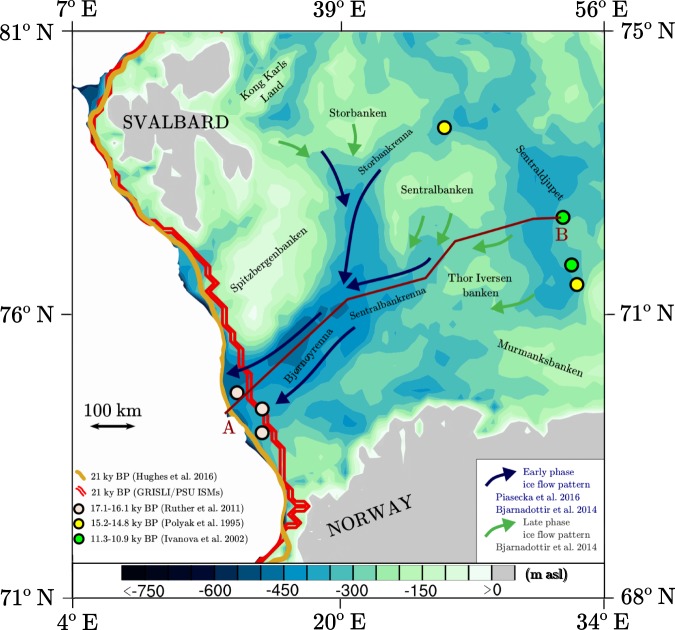
Bathymetric map of the Barents Sea (based on the International bathymetric chart of the Arctic Ocean (IBCAO)^27^ and shown here with the same horizontal resolution of 20 km used in the GRISLI ISM) with the reconstructed (DATED-1^1^, dark yellow line) and simulated (this study, red line) grounded ice margin position in the western Barents Sea during the LGM. Dark blue and grey arrows indicate the Bjørnøyrenna Ice Stream main ice flow patterns for early (dark blue) and late (grey) phases of ice streaming from observations^7,31^. Light grey and yellow circles indicate the position of the sediment cores providing the timing of the retreat of the Bjørnøyrenna Ice Stream from the outer shelf (white dots)^4^ and from Sentraldjupet (yellow dots)^5^. Green dots indicate the position of the sediment cores providing a time interval for ice-free conditions in Sentraldjupet^6^. The brown line (AB) represents the cross section of the Bjørnøyrenna Ice Stream analyzed in Figs 4 and 5.

The Eurasian Ice Sheet Complex during the Last Glacial Maximum (LGM, 21 ky BP) comprised three large ice sheets: the British-Irish Ice Sheet, the Fennoscandian Ice Sheet and the Barents Sea Ice Sheet^1^. In particular, the Barents Sea Ice Sheet was largely marine based, as its largest portion rested hundreds of meters below sea level on the Barents Sea floor. The Bjørnøyrenna Ice Stream was the largest ice stream draining the Barents Sea Ice Sheet^2^. At the LGM the ice stream extended up to the continental shelf edge in the western Barents Sea^3^ (Fig. 1). Sediment cores from the outer shelf in the south-western Barents Sea suggest that the Bjørnøyrenna Ice Stream started to retreat from the outer shelf to the inner part of Bjørnøyrenna between 17.1–16.1 ky BP^4^, (Fig. 1). Geological data show that the ice Stream had northern and eastern tributaries extending into the central Barents Sea, sourced from Storbanken/Storbankrenna and Sentralbankrenna/Sentralbanken respectively. Dates from glacimarine sediments suggest that the deglaciation of Sentraldjupet to the east of Bjørnøyrenna started between 15.2–14.8 ky BP^5^, and that ice-free conditions prevailed after 11.3–10.9 ky BP^6^ (Fig. 1).

Due to similar morphological and glaciological features, the Bjørnøyrenna Ice Stream is acknowledged as an analogue for several present-day ice streams in the West Antarctic Ice Sheet (WAIS)^7^. Therefore, it is interesting to identify the dynamic processes driving the retreat of the Bjørnøyrenna Ice Stream over the western Barents Sea continental shelf during the last deglaciation. Here, we focus on the role exerted by the sub-shelf melting and by grounding-line dynamics as triggers of the ice stream retreat. Several recent studies show that ice shelves and marine-terminating glaciers in the WAIS are thinning^8^ and retreating^9^ primarily due to sub-shelf melting occurring where relatively warm Circumpolar Deep Water reaches the ice-shelf cavities^10,11^. Analytical and numerical studies suggest that an initial retreat of the grounding-line position can lead to abrupt changes in volume and extent of marine-based ice sheets in regions where the bedrock is sloping towards the interior of the ice sheet (*i.e*., retrograde slopes)^12–14^. However, these studies analyzed the instability of marine-based ice sheets on retrograde slopes under conditions of one dimensional horizontal (1DH) flow^12–14^. When two dimensional horizontal (2DH) flow is considered, specific numerical examples of stable grounding lines on retrograde slopes have been simulated with various numerical Ice Sheet Models (ISMs)^15^. Nevertheless, several studies expanded and incorporated the 1DH boundary layer analytical solution proposed by Schoof^13^ for the flux across modelled grounding lines in large-scale three dimensional ISMs^16,17^. Therefore, the question of whether the WAIS will be susceptible to marine-ice sheet instability in the future remains unanswered, although geological evidence shows that the WAIS retreated frequently over the past 5 million years^18^.

In this study, we perform transient simulations with two ISMs, namely the GRenoble Ice Shelf and Land Ice model (GRISLI)^19^ and the Stockholm Branch of Penn State University glacial model (PSU)^20^, in order to investigate the role played by sub-shelf melting and grounding-line dynamics as triggers of the retreat of the Bjørnøyrenna Ice Stream after the LGM. The timing of the simulated retreat from Sentraldjupet is compared with deglacial chronologies derived from marine geological data^5,6^ (Fig. 1).

## Results and Discussion

### Evaluation of numerical models’ skill

**Figure 2:**
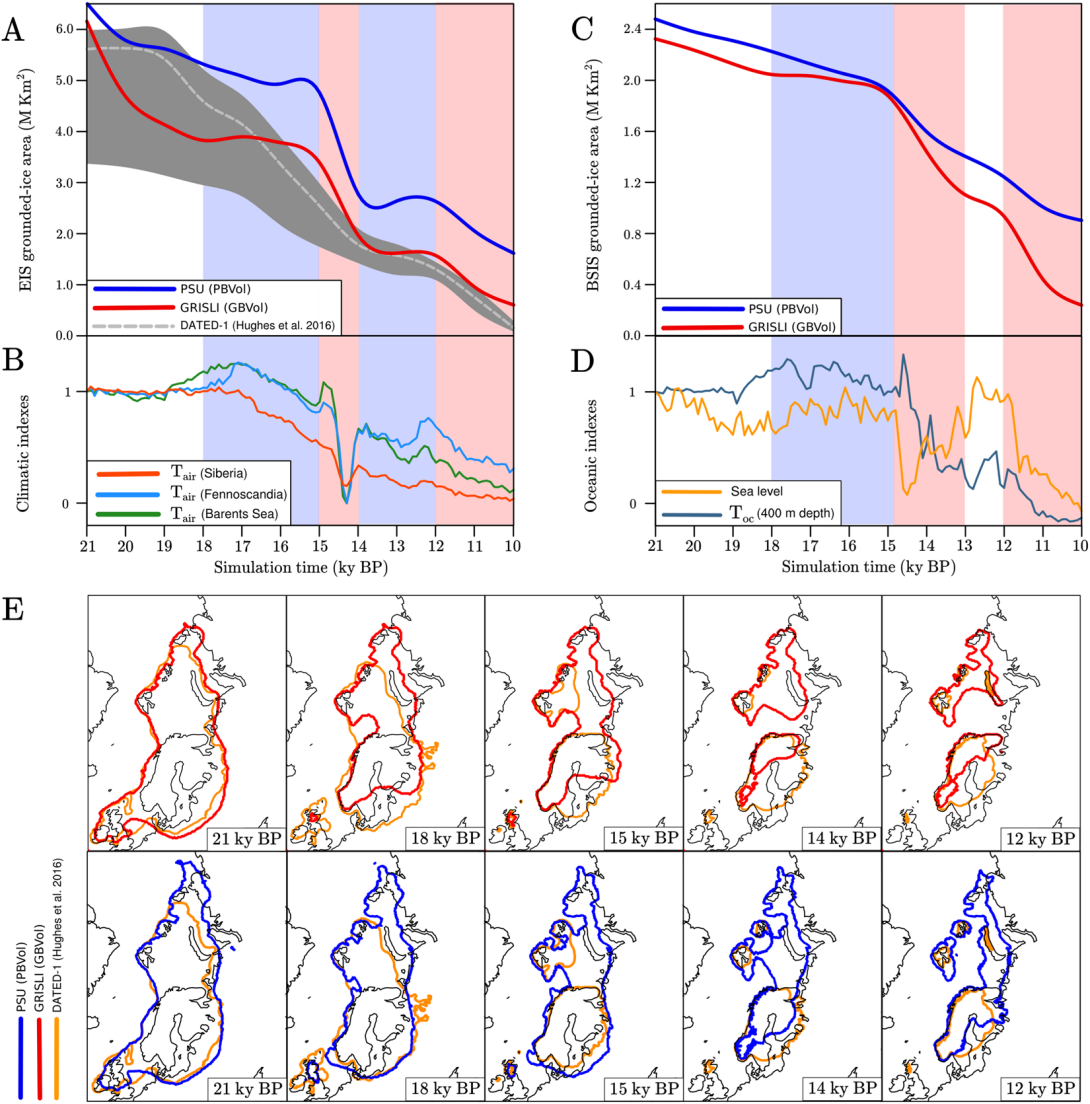
(**A**) Time evolution of the Eurasian Ice Sheet area in GBvol (red line) and PBvol (blue line) simulations. The Eurasian Ice Sheet area evolution during the deglaciation based on the DATED-1 reconstruction^1^ is indicated in dashed grey (maximum credibility) and shaded black (minimum and maximum credibility, respectively). Red/blue shades in the background highlight periods of warm/cold annual mean air temperature prescribed in the ISM simulations. (**B**) Mean annual air temperature indexes used to interpolate between LGM and Pre-Industrial (1850 a.d., PI) climate conditions in the ISM simulations (*Material and Methods*), based on the Trace-21ka fully coupled transient simulation^22^. Red/blue shades in the background highlight periods of warm/cold annual mean air temperature prescribed in the ISM simulations. (**C**) Time evolution of the Barents Sea grounded ice area in GBvol (red line) and PBvol (blue line) simulations. (**D**) Indexes for the annual mean ocean temperature in the South-western Barents Sea at 400 m depth (dark blue line) used to interpolate between LGM and PI ocean conditions and for the regional sea level (dark yellow line). The ocean temperature index is based on the Trace-21ka fully coupled transient simulation^22^, whereas the regional sea level index is based on the NGRIP *δ* 18 O record^29^. Red/blue shades highlight periods of warm/cold annual mean ocean temperature (400 m depth) and high/low global sea level prescribed in the ISM simulations. (**E**) Simulated extent of the Eurasian Ice Sheet in GBvol (red line, top panels) and PBvol (blue line, bottom panels) simulations at different time slices (21, 18, 15, 14 and 12 ky BP). The Eurasian Ice Sheet extent based on the DATED-1 reconstruction^1^ is shown in each panel in orange for comparison.

GRISLI and PSU best fit simulations (*Material and Methods*) GBvol and PBvol yield an Eurasian Ice Sheet grounded-ice area at the LGM of 6.5 and 6.1 10^6^ km^2^, respectively (Fig. 2A). The simulated LGM extent is slightly overestimated with respect to the data-based reconstruction DATED-1^1^, which suggests an LGM extent of 5.6 10^6^ km^2^ (Fig. 2A). Our modelled Barents Sea Ice Sheet eastern and northern margins as well as the eastern flank of the Fennoscandian Ice Sheet indeed expand further the DATED-1 reconstruction (Fig. 2E). We attribute these discrepancies to the climatology used to force the ISMs (*Material and Methods*). In our climate forcing, negative July and annual mean near-surface air temperatures occur over these regions at the LGM (*Supplementary Material*), while proxy data suggest higher air temperatures^21^.

**Figure 3:**
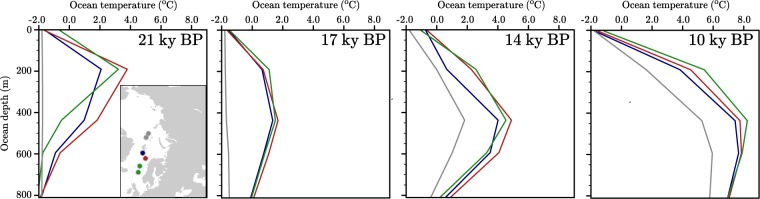
Ocean temperature vertical profiles derived from TraCE-21ka climate simulation^22^ at four different time slices (21, 17, 14, 10 ka). The vertical profiles are representative of the Norwegian Sea (green), South-Western Barents Sea (red), North-Western Barents Sea (blue) and Arctic Ocean (grey). In the inlay map, the points selected in the TraCE-21ka grid to derive the vertical profiles are shown.

Between 21 and 18 ky BP, a decrease in ice area of 2.30 and 1.20 10^6^ km^2^ is registered in GBvol and PBvol, respectively (Fig. 2A), whereas the DATED-1 reconstruction suggests a smaller decrease of about 1.00 10^6^ km^2^ (Fig. 2A). Compared to the DATED-1 reconstruction, in this early phase of the deglaciation the GRISLI ISM largely overestimates ice loss from the southern Fennoscandian and British-Irish ice sheets (2.00 10^6^ km^2^), whereas PBvol slightly overestimates ice loss from the British-Irish Ice Sheet (0.95 10^6^ km^2^, Fig. 2E). Since the prescribed annual mean air temperature remains close to its LGM value between 21 and 18 ky BP (Fig. 2B), the fast retreat is explained by the fact that in the GRISLI ISM most of the southern Fennoscandian Ice Sheet and the British-Irish Ice Sheet are treated with fast flow phyisics (ice velocity typically larger than 300 m/yr, *Supplementary Material*). This leads to to a massive ice transport towards the relatively warm, low-latitude southern margins of both ice sheets (*Supplementary Material*), where the ice is melted via surface ablation. In the case of the high-latitude marine-based Barents Sea Ice Sheet, the combined effect of sea-level rise (Fig. 2D) and sub-shelf melting (ocean temperature around 2 °C at the LGM, Fig. 3) triggers an initial retreat on the western margin in GBvol simulation, resulting in a decrease in ice area of 0.30 10^6^ km^2^ (Fig. 2C,E). The PBvol simulation shows a similar decrease in ice area (0.25 10^6^ km^2^, Fig. 2C) during the same time interval, although, in contrast to GBvol simulation, the ice retreat occurs on both the western and the northern margins of the Barents Sea Ice Sheet (Fig. 2E). The extremely cold ocean temperatures prescribed between 21 and 18 ky BP at the northern margin of the Barents Sea Ice Sheet (lower than 0 °C, Fig. 3) suggest that in PBvol the ice margin retreat might be triggered by internal ice dynamics rather than by sea-level rise and sub-shelf melting. The GRISLI ISM appears to be excessively sensitive to the combined effect of sea-level rise and sub-shelf melting in the western Barents Sea. Conversely, in the PSU ISM fast flowing ice and associated high ice discharge values at the grounding-line (typically more than one order of magnitude than surface ablation and sub-shelf melting) cause the retreat of all major ice streams in the western and northern Barents Sea in the early phase of the deglaciation.

Between 18 and 15 ky BP, both GBvol and PBvol simulations show a small decrease in ice area (0.44 and 0.56 10^6^ km^2^, respectively, Fig. 2A). The DATED-1 reconstruction exhibits a larger decrease of 2.10 10^6^ km^2^ (Fig. 2A), which is mostly due to the deglaciation of the Barents Sea Ice Sheet (Fig. 2E). However, the Barents Sea Ice Sheet retreat history suggested in the DATED-1 reconstruction is highly uncertain and based on a geological interpretation of limited chronological data from Novaya Zemlya and the eastern Barents Sea^1^. During this time interval, the Barents Sea Ice Sheet area loss is considerably lower in both GBvol and PBvol simulations (0.17 and 0.31 10^6^ km^2^, respectively, Fig. 2B), as the combination of cold ocean temperatures prescribed in the western Barents Sea at typical grounding-line depths (around 1.0 °C, Fig. 3) and nearly constant sea level (Fig. 2D) prevents extensive grounding-line retreat along the Barents Sea Ice Sheet western margin. Therefore, the larger ice loss simulated in the PBvol simulation with respect to the GBvol simulation comfirms that in the PSU ISM, sea-level rise and sub-shelf melting only play a secondary role compared to internal ice dynamics. Conversely, the high sensitivity of the GRISLI ISM to ocean temperatures and sea level has a stabilizing effect on the Barents Sea Ice Sheet during this time interval.

Between 15 and 14 ky BP, the rate of ice loss accelerates in both GBvol and PBvol simulations (ice area loss of 1.43 and 1.98 10^6^ km^2^, respectively, Fig. 2A), whereas the DATED-1 reconstruction suggests a reduction in ice area of 0.80 10^6^ km^2^ (Fig. 2A). In both simulations, the relatively warm annual mean air temperatures prescribed between 14.7 and 14 ky BP (corresponding to the Bølling-Allerød warming, Fig. 2B) cause an extensive retreat of the southern margin of the Fennoscandian Ice Sheet that is not supported by the DATED-1 reconstruction (Fig. 2E). The cause of such discrepancy in our simulations can be explained by the simplified method adopted here to compute the surface ablation (*Material and Methods*) which appears to be overly sensitive to atmospheric warming. Due to its high latitude location, the simulated Barents Sea Ice Sheet is less sensitive to atmospheric warming and its retreat is more reduced to that of the Fennoscandian Ice Sheet (Fig. 2E). However, the combined effect of abrupt sea-level rise and ocean warming occurring after 14.7 ky BP (Figs 2D and 3) causes a reduction in ice area in the Barents Sea Ice Sheet (Fig. 2E) which is higher in the GBvol simulation (0.44 10^6^ km^2^) than in the PBvol simulation (0.32 10^6^ km^2^).

Between 14 and 12 ky BP the Fennoscandian and the Barents Sea ice sheets show opposite behaviour in both GBvol and PBvol simulations. The cold annual mean air temperatures prescribed over the Fennoscandian Ice Sheet^22^ (corresponding to the Younger Dryas period, Fig. 2B) cause an expansion of the Fennoscandian Ice Sheet (Fig. 2E). In contrast, the Barents Sea Ice Sheet continues to retreat during this time interval (ice area loss of 0.50 and 0.36 10^6^ km^2^ in GBvol and PBvol simulations, respectively, Fig. 2C) as a result of warm ocean temperatures (Fig. 3), which overcomes the effect of the cold annual mean air temperatures simulated over the Barents Sea^22^ (Fig. 2D). The simulated opposite behaviour of the Fennoscandian Ice Sheet and Barents Sea Ice Sheet confirms that the simulated retreat of the marine-based Barents Sea Ice Sheet is mostly driven by oceanic forcing and grounding-line dynamics, rather than by atmospheric forcing. Between 12 and 10 ky BP, the combination of warm annual mean air temperatures over the Fennoscandian Ice Sheet^22^ (Fig. 2B), warm ocean temperatures in the Barents Sea (Fig. 3) and relatively high sea level (Fig. 2D) leads to the final retreat of the Eurasian Ice Sheet Complex (Fig. 2A) in both GBvol and PBvol simulations.

Recently, Patton *et al*.^23^ used a thermo-mechanical ISM adapted from the PSU ISM^24^ to simulate the retreat of the Eurasian Ice Sheet Complex after 23 ky BP. In Patton *et al*.^23^, the maximum ice sheet area is obtained at 22.7 ky BP (5.6 10^6^ km^2^)^23^ and is mostly in agreement with the DATED-1 reconstruction, except over Severnaya Zemlya, where the ice sheet is more expanded. In Patton *et al*.^23^ the evolution of the Fennoscandian and British-Irish ice sheets throughout the deglaciation is adequately simulated compared to the DATED-1 reconstruction, in spite of an overestimation of the area treated with fast flow physics in the southern Fennoscandian Ice Sheet, similarly to our GBvol simulation. Until 16 ky BP, the evolution of the Barents Sea Ice Sheet in Patton *et al*.^23^ is similar to the DATED-1 reconstruction. After this time, Patton *et al*.^23^ simulate a larger northern and eastern Barents Sea Ice Sheet than observed, although the discrepancy with the DATED-1 reconstruction is lower than that observed in GBvol and PBvol simulations. The better agreement obtained by Patton *et al*.^23^ with the DATED-1 reconstruction can be explained by the different approach used to force the ISMs in terms of atmospheric temperature, precipitation and ocean temperature. Although both studies use the same positive degree-day scheme to determine the surface mass balance over the ice sheet, Patton *et al*.^23^ tuned the regional reference climatologies and the associated climate forcings for the Fennoscandian, Barents Sea and British-Irish ice sheets in order to match a suite of empirical data^23^. Furthermore, in contrast to what is done in the present study, Patton *et al*.^23^ do not explicitly compute the sub-shelf melting. The retreat of the marine-terminating margins of the ice sheet is determined by an empirical function which relates calving to ice thickness and water depth^23^. Similarly as for the regional climate indexes, the sensitivity of the Fennoscandian, Barents Sea and British-Irish ice sheets to calving is independently tuned throughout the deglaciation^23^. In our study, climatic and oceanic forcings are derived from a transient climate simulation^22^ (*Material and Methods*) and are not modified. The two studies are therefore not directly comparable.

### Bjørnøyrenna Ice Stream evolution during the deglaciation

#### Grounding-line discharge and ice stream retreat

It emerges from our simulations that the retreat of the Barents Sea Ice Sheet is mostly driven by changes in ocean temperatures and sea level in the GBvol simulation. In contrast, in the PBvol simulation the ice sheet appears to be less sensitive to variations in ocean conditions and the major role in triggering the ice sheet retreat is played by grounding-line dynamics. In order to better establish the interplay between ocean warming, sea level and grounding-line physics in triggering the retreat of the Bjørnøyrenna Ice Stream, we focus on the evolution of its southern branch between 21–10 ky BP (cross section A–B, Fig. 1).

**Figure 4:**
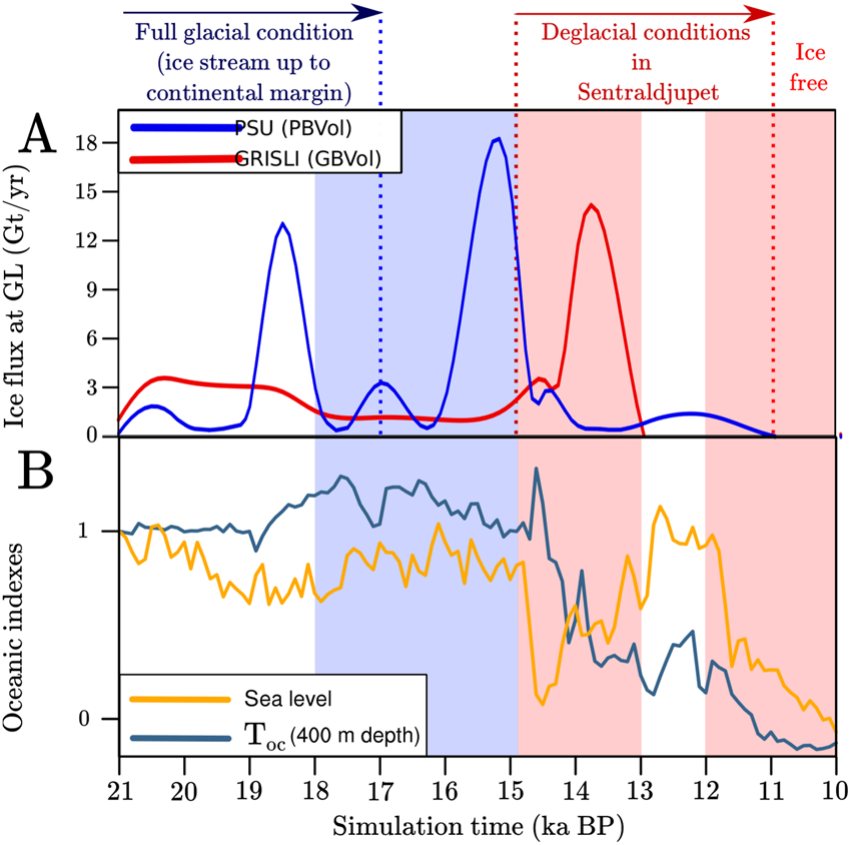
(**A**) Time evolution of the ice flux at the grounding-line along the Bjørnøyrenna Ice Stream cross section AB shown in Fig. 1 in GBvol (red line) and PBvol (blue line) simulations. When the ice flux at the grounding-line approaches zero, the Bjørnøyrenna Ice Stream can be considered as fully retreated. The Bjørnøyrenna Ice Stream retreat history according to geological data^4–6^ is summarized at the top of the plot. Red/blue shades highlight periods of warm/cold annual mean ocean temperature (400 m depth) and high/low global sea level prescribed in the ISM simulations. (**B**) Indexes for the annual mean ocean temperature in the South-western Barents Sea at 400 m depth (dark blue line) used to interpolate between LGM and PI ocean conditions and for the regional sea level (dark yellow line). The ocean temperature index is based on the Trace-21ka fully coupled transient simulation^22^, whereas the regional sea level index is based on the NGRIP *δ* 18 O record^29^. Red/blue shades highlight periods of warm/cold annual mean ocean temperature (400 m depth) and high/low global sea level prescribed in the ISM simulations.

Between 21 and 18 ky BP, ocean temperatures in the western Barents Sea remain nearly constant and are warm enough to cause sub-shelf melting (around 2 °C at typical grounding-line depths, Fig. 3). In the GBvol simulation, the ice flux at the grounding-line remains nearly constant around 3 Gt/yr during this time interval (Fig. 4), regardless of the sea-level rise prescribed between 20 and 18 ky BP. This suggests that in the GRISLI ISM sea-level rise alone cannot trigger massive ice discharges when it is not supported by ocean warming. In PBvol simulation the ice discharge at the grounding-line remains lower than 3 Gt/yr between 21 and 19 ky BP and abruptly peaks at 12 Gt/yr between 19 and 18 ky BP (Fig. 4). Since the initial sea-level rise prescribed in the simulation starts at 20 ky BP and the ocean temperatures remain nearly constant between 21 and 18 ky BP (Fig. 4), such a peak in ice discharge appears to be unrelated to oceanic conditions, thus confirming that in the PSU ISM the grounding-line dynamics exhert a primary role in triggering ice discharges at the margins of a marine-based ice sheet.

Between 18 and 15 ky BP, the ice discharge at the grounding-line remains lower than 3 Gt/yr in GBvol, likely in response to colder ocean temperatures than during the LGM (less than 2 °C at typical grounding-line depths, Fig. 3) and nearly constant sea level (Fig. 4). Conversely, the ice discharge simulated with the PSU ISM reaches its highest peak between 16 and 14.5 ky BP (around 18 Gt/yr, Fig. 4), which is, again, unrelated to changes in oceanic conditions. Interestingly, the peaks in ice discharge observed in PBvol simulation between 21 and 15 ky BP occur approximately every 1000 years and resemble the cyclic surging on millennial timescale simulated by Feldmann *et al*.^25^ for a marine ice-sheet-shelf system and driven by internal ice dynamics.

In the GBvol simulation, a relatively small peak in ice discharge (around 3 Gt/yr, Fig. 4) is observed at 14.7 ky BP, when a rapid sea-level rise is prescribed in correspondance with the Meltwater Pulse 1A (MWP-1A), which has been detected in sediment cores from the North-western Barents Sea^26^ (Fig. 4B). However, at that time the ocean temperature at typical grounding-line depths is still lower than 2 °C, which explains the low magnitude of the ice discharge. The largest ice discharge (about 14 Gt/yr) occurs around 14.5 ky BP and follows the ocean warming (around 4 °C at typical grounding-line depths, Fig. 3) simulated after 14.7 ky BP (Fig. 4), confirming the primary role of ocean temperatures in regulating ice mass loss in the GRISLI ISM. Conversely, in PBvol simulation the ice discharge remains lower than 3 Gt/yr between 15 and 13 ky BP, thus highlighting the low sensitivity of the grounding-line dynamics to variations in the sea level in the PSU ISM.

By 13 ky BP, the 400 m deep Sentraldjupet (Fig. 1) is fully deglaciated in GBvol (Fig. 4), whereas in PBvol the deglaciation of Sentraldjupet does not occur until 11 ky BP (Fig. 4). The 2,000 years delay between PBvol simulation and GBvol simulation can be explained by the different grounding-line physics implemented in the PSU ISM. The analytic ice flux imposed in the PSU ISM at each grounding-line grid node (or at the upstream grid node) depends on the ice thickness at the grounding line (*Material and Methods*). Therefore, when the simulated grounding-line is particularly thick, the outgoing ice flux computed with the solution by Schoof^13^ is very large compared to sub-shelf melting and ablation (typically more than one order of magnitude), thus dampening the effect of ocean warming on grounding-line retreat as observed in the GBvol simulation. As a result, the final deglaciation of Bjørnøyrenna Ice Stream in PBvol simulation is delayed with respect to the oceanic warming observed between 14.5 and 13 ky BP. Nevertheless, in both simulations the deglaciation timings are in agreement with the timing suggested by the geological data from the area^6^.

#### Response to ocean warming after Meltwater Pulse 1A

**Figure 5:**
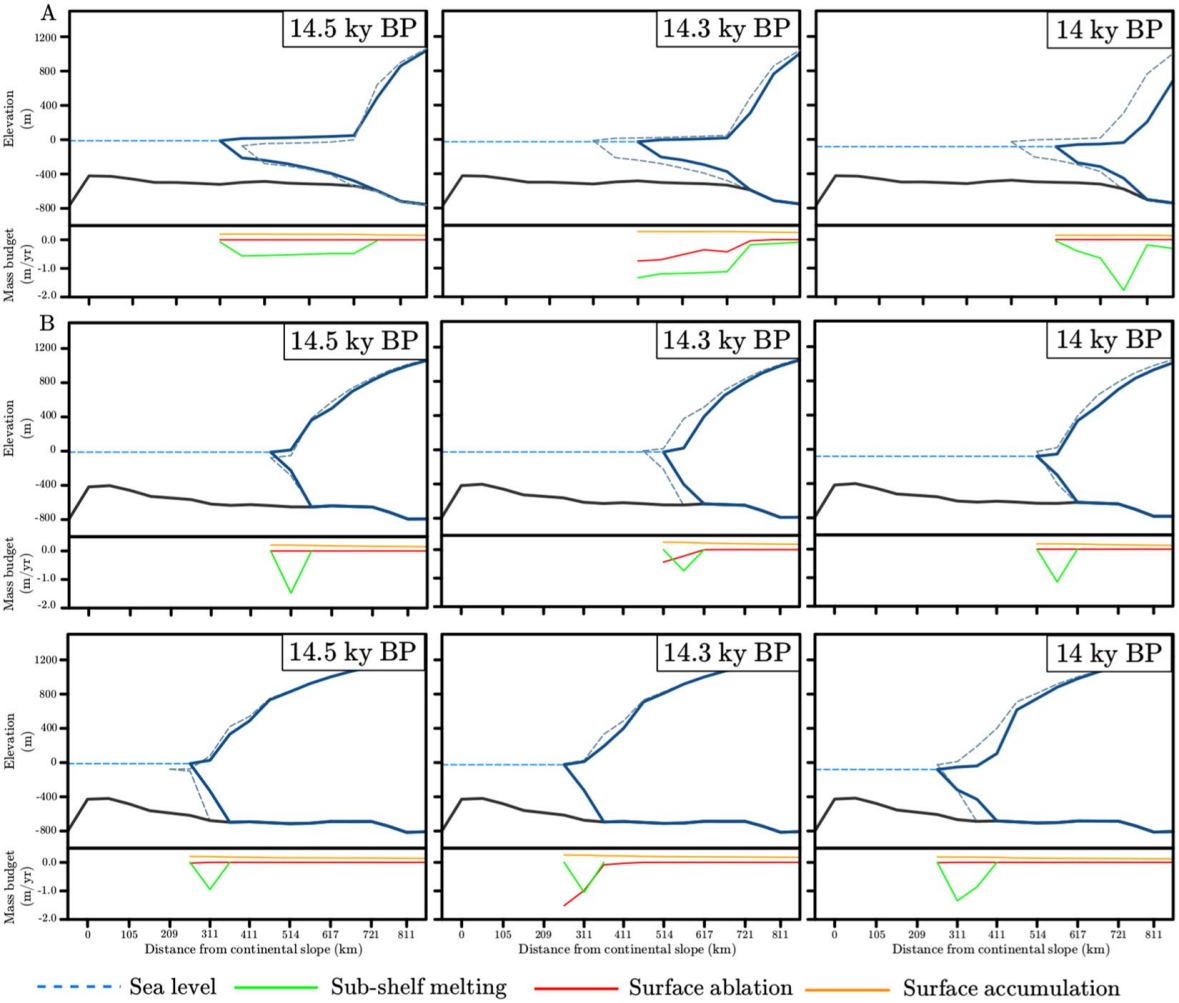
Profile of the Bjørnøyrenna Ice Stream along the cross section AB shown in Fig. 1 at 14.5, 14.3 and 14 ky BP (left, centre and right panel) respectively for (**A**) GBvol, (**B**) PBvol, (**C**) PBvolNS simulations. Continuous blue lines indicate the ice sheet elevation, whereas the continuous black and dashed light blue lines show the bedrock elevation and the regional sea level, respectively. The dashed grey lines indicate the ice sheet elevation at the previous time step to highlight the retreat scenario. Below each ice sheet profile, the mass budget (in meters per year) at each corresponding time frame is plotted: sub-shelf melting (green), surface ablation (red) and surface accumulation (orange) are shown.

In GBvol the abrupt sea-level rise occurring after 14.7 ky BP causes a grounding-line retreat of nearly 60 km (Fig. 5A). At 14.5 ky BP surface ablation is close to zero and ocean temperature at typical grounding line depths is still lower than 2 °C (Fig. 2D), thus resulting in a relatively low (around 0.5 m/yr) sub-shelf melting (Fig. 5A). As a consequence, the ice shelf advances about 40 km on the continental shelf (Fig. 5A). This suggests that the sea-level rise plays an important role in regulating the ice stream/shelf geometries, but has only a secondary effect compared to the sub-shelf melting and high grounding-line ice discharges, as shown in the previous section (Fig. 4). After 200 years, higher ocean temperatures at typical grounding-line depths (around 4 °C, Fig. 3) lead to increased sub-shelf melting (around 1.5 m/yr, Fig. 5A), which in turn causes a grounding-line retreat of about 20 km, consistent ice-shelf thinning and an ice-shelf front retreat of about 85 km (Fig. 5A). At 14 ky BP, sub-shelf melting reaches 2 m/yr (Fig. 5A), causing a grounding-line retreat of around 60 km into the Sentraldjupet and an ice-shelf front retreat of around 100 km (Fig. 5A).

In the PBvol simulation, MWP-1A does not affect the grounding-line position nor its thickness, despite relatively large sub-shelf melting of about 1.5 m/yr at 14.5 ky BP (Fig. 5B). At 14.3 ky BP, the grounding line retreats by 50 km in PBvol, despite a decrease in sub-shelf melting to 0.7 m/yr (Fig. 5B). In contrast, the increase in sub-shelf melting to about 1.2 m/yr at 14 ky BP has no effect on the grounding-line position nor on its thickness (Fig. 5B). This clearly confirms that the sub-shelf melting plays only a minor role in driving the ice stream retreat in PBvol simulation.

Since we forced both ISMs with the same vertical ocean temperature and salinity profiles, the reason for the different response of Bjørnøyrenna Ice Stream to sea-level rise and ocean warming in the GRISLI and PSU simulations can be attributed to the different ice stream/shelf geometries, which differ largely for both extent and thickness (Fig. 5A,B).

In order to understand the importance of the grounding-line flux correction by Schoof^13^ in determining the Bjørnøyrenna Ice Stream geometry and its resultant low sensitivity to variations in the oceanic conditions, we repeated the PBvol simulation by inhibiting the grounding-line flux correction in the PSU ISM (referred to as PBvolNS, *Material and Methods*). Without the grounding-line flux correction, the combined effect of sea-level rise and high sub-shelf melting (around 1.0 m/yr) at 14.5 ky BP does trigger a grounding-line retreat of 50 km in PBvolNS simulation (Fig. 5C), similarly to what is simulated in the GBvol simulation (Fig. 5A). The effect of the sub-shelf melting (1.0 m/yr) leads to ice thinning at the grounding line at 14.3 ky BP (Fig. 5C), which is followed by a 50 km grounding-line retreat at 14 ky BP, consistently with what is simulated with the GRISLI ISM (Fig. 5A). Moreover, at 14 ky BP (*i.e*., after sea-level rise and ocean warming) the ice stream/shelf geometries are similar to that simulated in GBvol, with a 100-km long and 400-m thick ice shelf (Fig. 5C). When no grounding-line flux correction is allowed in the PSU ISM, the simulated grounding-line position at 14 ky BP is 200 km closer to the continental slope than in the PBvol simulatiom (Fig. 5C). This demonstrates the large impact of the grounding-line flux formulation by Schoof^13^ on the simulated retreat history of the Bjørnøyrenna Ice Stream.

## Concluding Remarks

We have presented transient numerical simulations of the last deglaciation of the Eurasian Ice Sheet Complex, carried out with two different ISMs. Our aim was to evaluate the role played by sub-shelf melting and grounding-line dynamics in triggering the retreat of the Bjørnøyrenna Ice Stream, a palaeo-ice stream resting on a retrograde slope during the LGM and, therefore, considered to be a potential analogue for some contemporary WAIS ice streams^7^. The simulations suggest that in the GRISLI ISM, the decay of the marine-based portions of the Eurasian Ice Sheet Complex are mostly driven by variations in sea level and ocean temperatures rather than by variations in near-surface air temperatures. In particular, most of the grounded ice in the western Barents Sea retreats in response to a rapid change in the oceanic conditions occurring after 14.7 ky BP. For the Bjørnøyrenna Ice Stream, the combination of abrupt sea-level rise and ocean warming triggers a grounding-line retreat of 145 km in 700 years, which is followed by the final decay of the ice stream by 13 ky BP. The PSU ISM shows a lower sensitivity to variations in the oceanic conditions compared to the GRISLI ISM, which we attribute to implementation of the grounding-line flux correction of Schoof^13^. We then demonstrate that large grounding-line fluxes computed with the solution by Schoof^13^ can reduce the importance of the sub-shelf melting on grounding-line retreat. When the grounding-line flux correction is inhibited, the PSU ISM shows a response to sea-level rise and ocean warming similar to those simulated with the GRISLI ISM. These findings suggest that the use of 1DH flow-line solutions for the grounding-line flux in 3D numerical models could be problematic^15^. In order to provide reliable quantitative estimates of the WAIS contribution to future variations in the sea level or in the ocean temperature, a better comprehension and representation of the dynamical processes acting at the grounding-line is needed.

## Material and Methods

The numerical ISMs employed are the Stockholm Branch of PSU^20^ and GRISLI^19^. Both ISMs are hybrid shallow ice/shallow shelf approximation models, able to simulate grounded (ice sheets and ice streams) and floating ice (ice shelves). As opposed to the GRISLI ISM, the 1DH solution from^13^ is imposed as a condition on the ice flux across the grounding line in the PSU ISM. The ISMs’ dynamics is fully described in^19^ and^20^, whereas the boundary conditions and a list of model parameter values are included in the *Supplementary Material*. All the GRISLI/PSU simulations were performed using a horizontal resolution of 20 km/18.5 km, respectively. With both ISMs, transient spin-up simulations were run between 122 ky BP (MIS5) and the LGM, in order to initialize the thermodynamical state of the Eurasian Ice Sheet. At the beginning of each spin-up simulation, a masked ice-free present-day surface topography based on the IBCAO chart^27^ and the PI annual mean air temperature and precipitation simulated with the IPSL-CM5A-LR^28^ AOGCM were prescribed (*Supplementary Material*). During the spin-up simulations, the climatology was progressed from PI to the LGM by means of a climate index based on the NGRIP *δ* 18 O record^29^. At the LGM, the annual mean air temperature and precipitation simulated by the IPSL-CM5A-LR^28^ model were prescribed (*Supplementary Material*). The PI climate fields were downscaled on the ISM grids by means of the present-day surface topography based on the IBCAO chart^27^. The LGM surface topography based on the ICE-5G^30^ glacio-isostatic reconstruction was used to downscale the LGM climate fields. A low constant sub-shelf melting rate was prescribed during all spin-up simulations in order to allow the Barents Sea Ice Sheet to spread over the Barents and Kara Seas (*Supplementary Material*). In the transient simulations of the last deglaciation the downscaled LGM and PI annual mean air temperature and precipitation simulated with the IPSL-CM5A-LR^28^ model were prescribed as initial and final climate snapshots, respectively, with both ISMs. During the simulations, the climatology was progressed from the LGM to PI by means of three different climate indexes for annual mean air temperature and precipitation derived from TraCE-21ka, a non-accelerated transient climate simulation of the last 21 ka^22^. The three different indexes for annual mean air temperature and precipitation were taken as representative of Fennoscandia, Svalbard/Barents Sea and Siberia/Kara Sea macro-regions (Fig. 2B and *Supplementary Material*). During the transient simulations of the last deglaciation, the sea level was progressed from −130 to 0 m following an index based on the NGRIP *δ* 18 O record^29^ (Fig. 2D). In both the GRISLI and PSU ISMs, sub-shelf melting under the ice shelves is computed following the two-equations formulation adopted in^17^. During the transient simulations of the last deglaciation, the sub-shelf melting formulation was forced with four time-varying ocean temperature and salinity profiles derived from TraCE-21ka^22^. The four different ocean temperature and salinity vertical profiles were taken as representative of the Norwegian Sea, the South-western and North-western Barents Sea and the Arctic Ocean, respectively (*Supplementary Material*). With the GRISLI ISM a Latin Hypercube Sampling (LHS) of five model parameters was constructed (*Supplementary Material*) and a statistical ensemble of 101 transient simulations of the last deglaciation was generated. The Eurasian Ice Sheet Complex ice volume evolution during the deglaciation simulated with the GRISLI ISM was compared with those simulated with the global glacio-isostasy model ICE-5G^30^, and a best fit simulation (referred to as GBvol) from the statistical ensemble was identified (*Supplementary Material*). Due to large computational costs, it was not possible to pursue the same statistical approach with the PSU ISM. Therefore, the optimal parameter values identified with the GRISLI ISM were used to run a corresponding PSU best fit transient simulation (referred to as PBvol, *Supplementary Material*). In order to investigate the impact of the grounding-line flux correction^13^ implemented in the PSU ISM on the retreat of the Bjørnøyrenna Ice Stream, the simulation PBvol was repeated inhibiting the grounding-line correction (referred to as PBvolNS).

## Electronic supplementary material

Supplementary Material
