# Author Correction: Interplay of grounding-line dynamics and sub-shelf melting during retreat of the Bjørnøyrenna Ice Stream

**DOI:** 10.1038/s41598-018-30242-x

**Published:** 2018-08-21

**Authors:** Michele Petrini, Florence Colleoni, Nina Kirchner, Anna L. C. Hughes, Angelo Camerlenghi, Michele Rebesco, Renata G. Lucchi, Emanuele Forte, Renato R. Colucci, Riko Noormets

**Affiliations:** 1 OGS (Istituto Nazionale di Oceanografia e Geofisica Sperimentale), Borgo Grotta Gigante 42/c, 34010 Sgonico, TS Italy; 2 CMCC (Centro Euro-Mediterraneo sui Cambiamenti Climatici), via Franceschini 31, 40128 Bologna, BO Italy; 3 Bolin Centre for Climate Research, Stockholm University, SE-106 91 Stockholm, Sweden; 4 Department of Earth Science, University of Bergen and Bjerknes Centre for Climate Research, N-5020 Bergen, Norway; 5 Dipartimento di Matematica e Geoscience, Universit di Trieste, Piazzale Europa 1, 34127 Trieste, TS Italy; 6 ISMAR (Istituto di Scienze Marine), Trieste, Italy; 7 The University Centre in Svalbard (UNIS), P.O. Box 156 Northern-9171, Longyearbyen, Norway; 8 Present Address: Department of Geoscience and Remote Sensing, Delft University of Technology (TUDelft), Delft, Netherlands

Correction to: *Scientific Reports* 10.1038/s41598-018-25664-6, published online 08 May 2018

The Acknowledgements section in this Article is incomplete.

“This work was funded by the National Institute of Oceanography and Applied Geophysics (OGS), by the University of Trieste (UNITS) and by the FORMAS grant 214-2013-1600 to Nina Kirchner. We acknowledge the CINECA award under the ISCRA initiative, for the availability of high performance computing resources and support. Parts of the computations were performed on resources provided by the Swedish National Infrastructure for Computing (SNIC) at PDC Center for High Performance Computing at KTH. The authors would like to thank the anonymous reviewers for their valuable comments and suggestions to improve the quality of the paper.”

should read:

“The research reported in this work was supported by Oceanography and Applied Geophysics (OGS) and CINECA under HPC-TRES program award number 2016-03, by University of Trieste (UNITS) and by the FORMAS grant 214-2013-1600 to Nina Kirchner. We acknowledge the CINECA award under the ISCRA initiative, for the availability of high performance computing resources and support. Parts of the computations were performed on resources provided by the Swedish National Infrastructure for Computing (SNIC) at PDC Center for High Performance Computing at KTH. The authors would like to thank the anonymous reviewers for their valuable comments and suggestions to improve the quality of the paper.”

