# Supplementary material for: Interplay of grounding-line dynamics and sub-shelf melting during retreat of the Bjørnøyrenna Ice Stream
Source: Sci Rep. 2018 May 8;8:7196. doi: 10.1038/s41598-018-25664-6 (PMC5940849; doi:10.1038/s41598-018-25664-6)
Supplement: Supplementary file 1 — Supplementary Material [file 41598_2018_25664_MOESM1_ESM.pdf]

Interplay of grounding-line dynamics and sub-shelf melting during retreat of the Bjørnøyrenna Ice Stream

Michele Petrini, Florence Colleoni, Nina Kirchner, Anna L.C. Hughes, Angelo Camerlenghi, Michele Rebesco, Renata G. Lucchi, Emanuele Forte, Renato R. Colucci, and Riko Noormets

This supplementary material contains the following:

- Description of the climate forcing in the ice sheet simulations;
- Description of the surface mass balance scheme employed in the ice sheet simulations;
- Description of the method employed to force the ocean basal melting parametrization from Pollard & DeConto (2012) in the ice sheet simulations;
- Description of the statistical approach used in this study to set the parameters for GRISLI and PSU models.

Air temperature and precipitation forcing

TraCE-21ka transient climate simulation Liu et al. (2009) is used to construct different indexes for air temperature and precipitation representative of Fennoscandia, Svalbard/Barents Sea and Siberia/Kara Sea macro-regions. For each macro-region, seven different nodes in the TraCE-21ka grid are selected (Fig.1g-h-i). In each grid node, annual mean air temperature T_a and annual mean precipitation P_a are computed every 100 years from the Last Glacial Maximum (around 21,000 years ago, LGM) to PI (1850 a.d., PI), as shown in Fig.1a-f. In each macro-region, the average annual mean near-surface air temperature $\overline{T}_c^a$ and precipitation $\overline{P}_c^a$ are considered for each macro-region and at each time step (Fig.1a-f). For each macro-region and at each time step i, the temperature and precipitation indexes I_T and I_P are computed as follows,

$$I_T(i) = \frac{\overline{T}_c^a(i) - \overline{T}_c^a(\mathrm{PI})}{\overline{T}_c^a(\mathrm{LGM}) - \overline{T}_c^a(\mathrm{PI})}\,, \tag{1}$$

$$I_P(i) = \frac{\overline{P}_c^a(i) - \overline{P}_c^a(\mathrm{PI})}{\overline{P}_c^a(\mathrm{LGM}) - \overline{P}_c^a(\mathrm{PI})}\,, \tag{2}$$

so that the all the indexes take value 1 at LGM and 0 at PI.

Surface mass balance

In both GRISLI and PSU ISMs the surface mass balance takes into account accumulation and ablation. The snow accumulation is computed from the annual mean total precipitation following Marsiat (1994). A linear transition between solid and liquid precipitation depending on the annual mean near-surface air tem-

perature is considered to compute the solid precipitation fraction P_{sf},

$$P_{\mathsf{sf}} = \begin{cases} 1 & \text{if } T \leq -10\,^{\circ}\mathrm{C}\,, \\ (7\,^{\circ}\mathrm{C} - T)\,/17\,^{\circ}\mathrm{C} & \text{if } -10\,^{\circ}\mathrm{C} < T \leq 7\,^{\circ}\mathrm{C}\,, \\ 0 & \text{if } T > 7\,.^{\circ}\mathrm{C} \end{cases} \tag{3}$$

The accumulation is then computed from the annual mean total precipitation as follows,

$$\mathsf{ACC} = P^a \cdot P_{\mathsf{sf}}\,. \tag{4}$$

The ablation is computed using the Positive-Degree-Days (PDD) semi-empirical method Reeh (1989). The PDD method relates the number of days with positive near-surface air temperature with snow and ice melting rates through observation-based melting coefficients. First of all, the near-surface air temperature is assumed to vary sinusoidally over time. Such an assumption allows to reconstruct the annual near-surface air temperature cycle from the annual and July mean near-surface air temperature,

$$T^m\,(t) = T^a + \left(T^j - T^a\right)\cos\frac{2\pi t}{A}\,, \tag{5}$$

where $A = 1\,\mathrm{year}$. Following Reeh (1989), the number of PDD is then computed from the normal probability distribution around the monthly mean temperatures during the year, *i.e.*,

$$\mathsf{PDD}_{\triangle\sigma} = \int_0^A \int_0^\infty \frac{1}{\sigma(t)\sqrt{2\pi}} T \exp\left(\frac{\left(T - T^m(t)\right)^2}{2\sigma^2(t)}\right) \mathrm{d}T\mathrm{d}t\,. \tag{6}$$

where t is the time variable, T (°C) is the near-surface air temperature variable, and σ is the standard deviation of the near-surface air temperature. In this study, the altitude-latitude dependent parametrization for the near-surface air temperature standard deviation proposed by Fausto et al. (2011) is used. Based on near-surface air temperature observations at the automatic weather stations on the Greenland Ice Sheet, annual and July mean standard deviations (σ^a and σ^j respectively) of the near-surface air temperature are parametrized as follows,

$$\sigma^a = 0.324 + 0.0011 \cdot s + 0.0573 \cdot \phi \tag{7}$$

$$\sigma^j = 2.22 + 0.0013 \cdot s - 0.0178 \cdot \phi\,, \tag{8}$$

where ϕ is the latitude expressed in °N. In such a way, in each grid point of the Eurasian Ice Sheet Complex domain mean annual and July standard deviation of near-surface air temperature are computed depending on the surface elevation and latitude. Then, similarly as for the near-surface air temperature (see Equation 5), a sinusoidally variation of the near-surface air temperature standard deviation over the year is assumed,

$$\sigma(t) = \sigma^a + \left(\sigma^j - \sigma^a\right)\cos\frac{2\pi t}{A}\,. \tag{9}$$

Once the number of PDD is calculated, the amount of snow and ice melted is related to the number of PDD by means of snow and ice melt coefficients (C_s and C_i respectively) depending on the annual mean near-surface air temperature Tarasov & Peltier (2002),

$$C_i = \begin{cases} C_i^c & \text{if } T^a < T^c\,, \\ C_i^w + \left(\frac{T^w - T^a}{T^w - T^c}\right)^3 \cdot (C_i^c - C_i^w) & \text{if } T^c \leq T^a < T^w\,, \\ C_i^w & \text{if } T \geq T^w\,, \end{cases} \tag{10}$$

$$C_s = \begin{cases} C_s^c & \text{if } T^a < T^c\,, \\ C_s^w + \left(\frac{T^a}{T^w - T^c}\right) \cdot (C_s^c - C_s^w) & \text{if } T^c \leq T^a < T^w\,, \\ C_s^w & \text{if } T \geq T^w\,, \end{cases} \tag{11}$$

where $T_c = -1\,^{\circ}\mathrm{C}$, $T_w = 10\,^{\circ}\mathrm{C}$, $C_i^c = 17.22\,\mathrm{mm\,d^{-1}\,^{o}C}$, $C_i^w = 8.3\,\mathrm{mm\,d^{-1}\,^{o}C}$, $C_s^c = 2.65\,\mathrm{mm\,d^{-1}\,^{o}C}$ and $C_s^w = 4.3\,\mathrm{mm\,d^{-1}\,^{o}C}$.

Once values for snow and ice melting coefficients are assigned, ablation takes place in three steps:

- fresh snow, if present, is melted first at rate C_s. Meltwater resulting from the melted snow is able to percolate into the snow layer and refreeze as superimposed ice. If the amount of superimposed ice exceeds 60% of the annual snowfall, runoff occurs;
- when all the snow is melted, superimposed ice, if present, is melted at rate C_i;
- when all the snow and the superimposed ice are melted, glacier ice is melted at rate C_i.

Depending on the snow accumulation and on the available melting energy in each time step, not all the steps (or even none of the steps) will be carried out, Colleoni et al. (2014).

Ocean basal melting forcing and parameter tuning

In order to force the ocean basal melting parametrization from Pollard & DeConto (2012), we derived from TraCE-21ka transient climate simulation Liu et al. (2009) four different vertical profiles of ocean temperature and salinity, respectively, which vary every 100 years from the LGM to PI and are representative of four macro-regions (Norwegian Sea, South-Western and North Western Barents Sea, Arctic Ocean, Fig.2, 8). For each macro-region, the ocean temperature and salinity profiles are included both in GRISLI and PSU numerical models in the following way:

- first, the LGM and PI ocean temperature and salinity at five selected vertical levels ($0\,\mathrm{m}$, $200\,\mathrm{m}$, $400\,\mathrm{m}$, $600\,\mathrm{m}$, $800\,\mathrm{m}$) are considered. The vertical levels are selected depending on typical Barents Sea depths. At each vertical level, the LGM and PI ocean temperature and salinity are obtained from TraCE-21ka simulation and included in GRISLI and PSU. Given a vertical level j,

LGM and PI ocean temperature and salinity are denoted with T^j_{LGM}, T^j_{PI}, S^j_{LGM} and S^j_{PI} respectively. At a given depth z_0, the ocean temperature and salinity profiles at LGM and PI are computed by linearly interpolating between the two vertical levels including z_0.

- at each vertical level j, an index for ocean temperature (i^j_T) and salinity (i^j_S), respectively, is computed every 100 years from TraCE-21ka data. At a given time t the temperature and salinity index, respectively, is given by

$$i^j_T = \frac{T^j(t) - T^j_{\mathrm{PI}}}{T^j_{\mathrm{LGM}} - T^j_{\mathrm{PI}}}\,, \tag{12}$$

$$i^j_S = \frac{S^j(t) - S^j_{\mathrm{PI}}}{S^j_{\mathrm{LGM}} - S^j_{\mathrm{PI}}}\,. \tag{13}$$

  In such a way all the indexes take value 1 at LGM and 0 at PI. All the ocean temperature and salinity indexes are included in GRISLI and PSU.

During a GRISLI or PSU simulation, given an ice shelf grid point the ISM identifies first the macro-region in which the point is contained depending on its longitude/latitude. The depth of the ice shelf base z_b is then computed, and the vertical levels j and $j+1$ such that $j \leq z_b < j+1$ are identified, where $j = 0,\ 200,\ 400,\ 600,\ 800$. At a given time t, the ocean temperature and salinity at vertical levels j and $j+1$ are computed as follows,

$$T^j(t) = i^j_T(t) \cdot T^j_{\mathrm{LGM}} + \left(1 - i^j_T\right) \cdot T^j_{\mathrm{PI}}\,, \tag{14}$$

$$T^{j+1}(t) = i^{j+1}_T(t) \cdot T^{j+1}_{\mathrm{LGM}} + \left(1 - i^{j+1}_T\right) \cdot T^{j+1}_{\mathrm{PI}}\,, \tag{15}$$

$$S^j(t) = i^j_S(t) \cdot S^j_{\mathrm{LGM}} + \left(1 - i^j_S\right) \cdot S^j_{\mathrm{PI}}\,, \tag{16}$$

$$S^{j+1}(t) = i^{j+1}_S(t) \cdot S^{j+1}_{\mathrm{LGM}} + \left(1 - i^{j+1}_S\right) \cdot S^{j+1}_{\mathrm{PI}}\,. \tag{17}$$

By linearly interpolating between $T^j(t)$, $S^j(t)$ and $T^{j+1}(t)$, $S^{j+1}(t)$ the ocean temperature and salinity at depth z_b are computed and used to force the ocean basal melting parametrization from Pollard & DeConto (2012).

Once an adequate oceanic forcing is set, a new strategy to tune the model parameter F_m is needed. In fact, the model parameter F_m has been hand-tuned in Martin et al. (2011) in order to match simulated and observed grounding-line position in the West Antarctic Ice Sheet. Therefore, the oceanic conditions used to force the ocean basal melting parametrization are drastically different from those derived from TraCE-21ka climate simulation and used in this study (Fig.2 and 8). An a-priori analysis of the oceanic forcing used in this study is performed, in order to identify a range of values for F_m. First of all, a typical grounding-line depth of 400 m in the north-western Barents Sea is considered. Looking at TraCE-21ka simulation, at such depth the warmer water temperature (around 7.5 °C, see Fig.2) is reached around 10 ka BP in the south-western and north-western Barents Sea. A water salinity of around 35.5 psu is reached at the same depth and time frame in the south-western and north-western Barents Sea. By forcing the basal melting parametrization from Pollard & DeConto (2012) with such "warm" ocean

conditions we obtain the maximum ocean basal melting rate between LGM and PI ($\mathsf{b_{max}}$) as a function of the model parameter F_m only, *i.e.*,

$$\mathrm{b}_{\mathsf{max}} = 41.97 \cdot F_m \cdot (9.73)^2 \ . \tag{18}$$

In such a way it is possible to identify a range of values for the model parameter F_m so that b_{max} matches the ocean basal melting values observed under the present-day Antarctica ice shelves. Estimates for the annual basal mass-loss rates of Antarctic ice shelves from Depoorter et al. (2013) and Rignot et al. (2013) range between $0.1 - 22\,\mathrm{m/yr}$. However, the averaged annual basal mass-loss rate of ice shelves in the West Antarctica Peninsula are lower $0.8-1.0\,\mathrm{m/yr}$, Depoorter et al. (2013). Therefore, we decided to set a range for b_{max} between $0.1 - 6\,\mathrm{m/yr}$. In such a way, the range identified for the model parameter F_m is

$$0.02 \cdot 10^{-3} \leq F_m \leq 1.56 \cdot 10^{-3} \, .$$

Latin Hypercube Sampling of model parameters

This statistical approach was previously used by Stokes & Tarasov (2010) to simulate the Laurentide Ice Sheet and Applegate et al. (2015) to simulate the Greenland Ice Sheet. In this study five GRISLI model parameters are considered for the Latin Hypercube Sampling (see Table 3):

- the topographic lapse-rate λ, which represents an approximation of how much the near-surface air temperature changes with elevation. This parameter is poorly constrained in large-scale ice sheet modeling studies, whereas topographic lapse-rate values computed from climate simulations range from 4 to $7\,^{\circ}\mathrm{C/km}$ Abe-Ouchi et al. (2007). Following Stone et al. (2010) and Colleoni et al. (2016), in this study the range 4 to $8.2\,^{\circ}\mathrm{C/km}$ is explored;
- the precipitation-correction factor γ, which approximates the saturation pressure of water vapour Charbit et al. (2002). In large-scale ice sheet modeling studies this parameter ranges between 0.03 and $0.078\,^{\circ}\mathrm{C}^{-1}$ (*e.g.*, Charbit et al. (2002)), although climate modelling studies suggest that γ assumes higher values up to $0.11\,^{\circ}\mathrm{C}^{-1}$. Following Colleoni et al. (2016), in this study the range $0.03 - 0.1\,^{\circ}\mathrm{C}^{-1}$ is explored;
- the SIA-enhancement factor $\mathsf{E}_{\mathrm{SIA}}$, accounting for the anisotropy of polycristalline ice under condition of simple-shear flow, Ma et al. (2010). In large-scale ice sheet modeling studies this parameter is set to values ranging from 1 to 5 (Colleoni et al. (2016) and references therein). However, an higher value of 5.6 is suggested by Ma et al. (2010) in a study where an anistropic full-Stokes model is used, thus explicitly accounting for grain orientation (fabric). Therefore, in accord with Colleoni et al. (2016) the range $1 - 5.6$ is explored;
- the basal drag coefficient c_f, which regulates the resistive force acting at the base of the ice sheet in ice stream regions treated with the shallow-shelf

approximation. In Wekerle et al. (2016) the strong impact of this parameter on the GRISLI-simulated ice thickness is shown. This parameter is set in early works with GRISLI to $1 \cdot 10^{-5}$ in Peyaud et al. (2007), to $9 \cdot 10^{-5}$ in Dumas (2002) and between $10 \cdot 10^{-5} - 100 \cdot 10^{-5}$ in Álvarez Solás et al. (2011). In this study the range $1 \cdot 10^{-5}$-$10 \cdot 10^{-5}$ is explored;

- the ocean basal melting parameter F_m the ocean basal melting parametrization from Pollard & DeConto (2012). In the previous section we show the approach adopted to set a range for this parameter. The range explored in this study is $0.02 \cdot 10^{-3}$-$1.56 \cdot 10^{-3}$.

Following this statistical approach, a group of 101 simulations is performed with GRISLI. The Eurasian Ice Sheet Complex ice volume evolution during the deglaciation simulated with GRISLI is compared with those simulated with the global glacio-isostasy model ICE-5G Peltier (2004), and a best fit simulation (referred to as GBvol) out of the statistical ensemble is identified. We did not use ICE-6G to constrain our ice sheet simulations since some flaws have been recently found in the more recent ICE-6G Peltier et al. (2015) reconstruction Purcell et al. (2016). Due to large computational costs, a similar statistical approach was not feasible with PSU. However, GRISLI and PSU have in common four of the five model parameters that are explored with GRISLI LHS, namely λ, γ, $\mathsf{E}_{\mathrm{SIA}}$ and F_m. As concerns the SIA-enhancement factor $\mathsf{E}_{\mathrm{SIA}}$, in PSU an high value of $\mathsf{E}_{\mathrm{SIA}} = 10$ is necessary to match the Hughes et al. (2016) reconstructed grounding-line position at the LGM in Bjørnøyrenna Trough. Such high value is out of the range explored with the LHS with GRISLI. In contrast, the optimal values for λ, γ and F_m employed in GBvol are employed to run a corresponding PSU best fit transient simulation, referred to as PBvol. Table 3 shows the parameter values in the best fit transient simulations GBvol and PBvol.

Tables and Figures

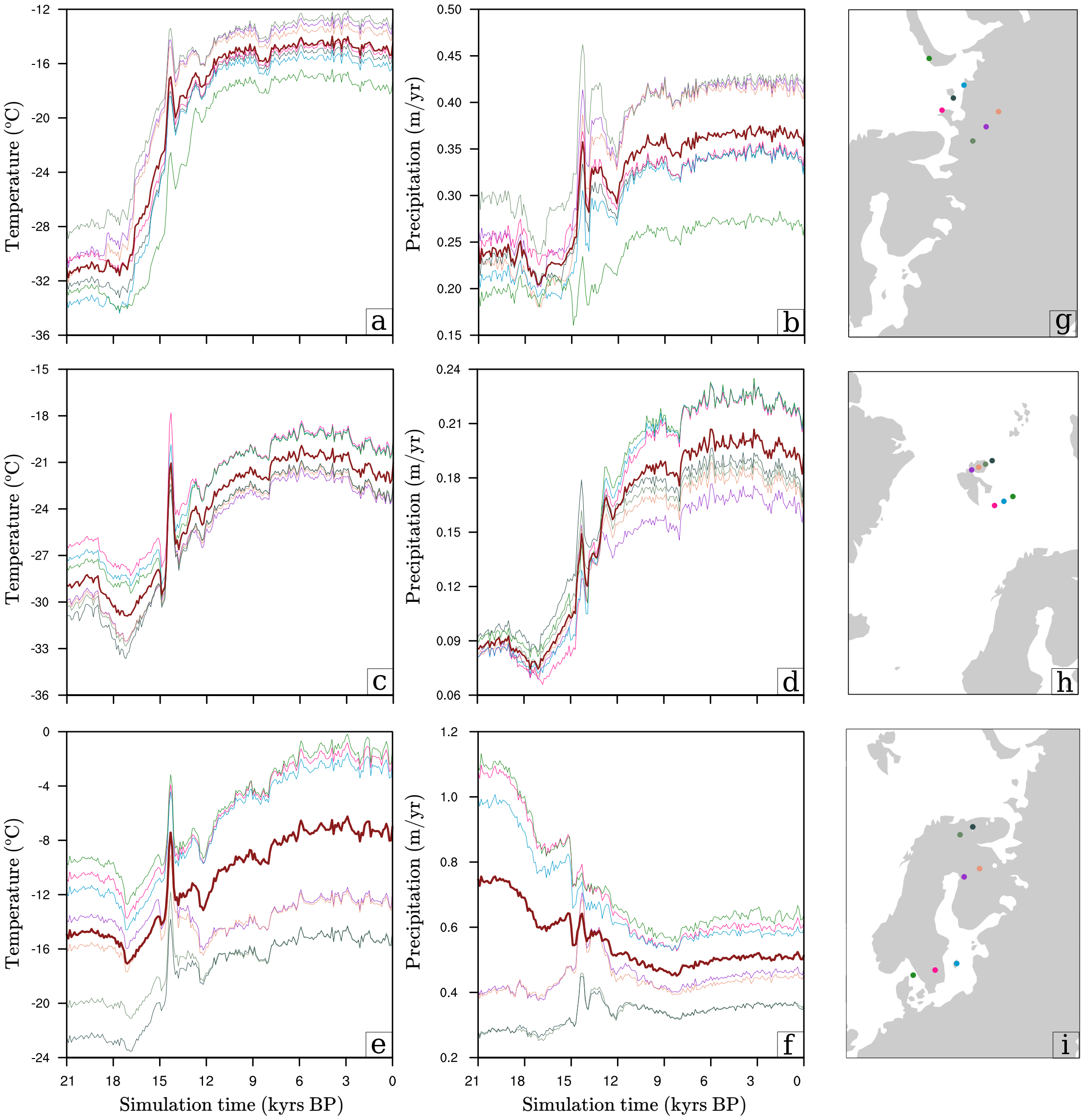

Figure 1: Annual mean near-surface air temperature and precipitation evolution at different TraCE-21ka grid points in the three macro-regions Siberia/Kara Sea (a)-(b), Svalbard/Barents Sea (c)-(d) and Fennoscandia (e)-(f). In dark red the average between the different grid points values is shown, whereas the location of the grid points in each macro-region is displayed in panels (g)-(h)-(i).

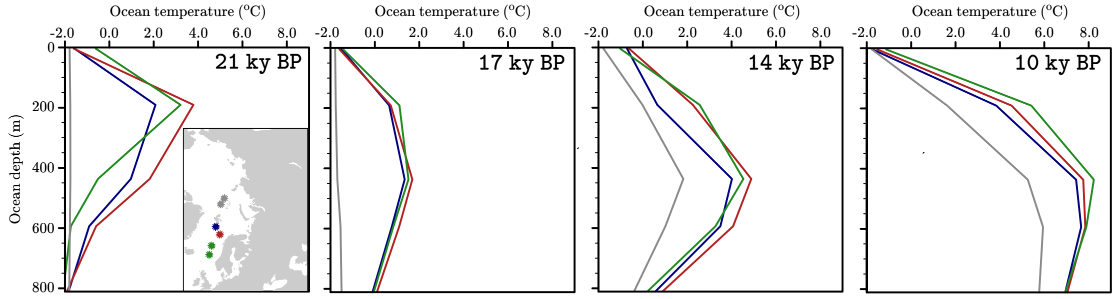

Figure 2: Ocean temperature vertical profiles derived from TraCE-21ka climate simulation Liu et al. (2009) at four different time slices (21, 17, 14, 10 ka). The vertical profiles are representative of the Norwegian Sea (green), South-Western Barents Sea (red), North-Western Barents Sea (blue) and Arctic Ocean (grey). In the inlay map the points selected in the TraCE-21ka grid to compute the vertical profiles are shown.

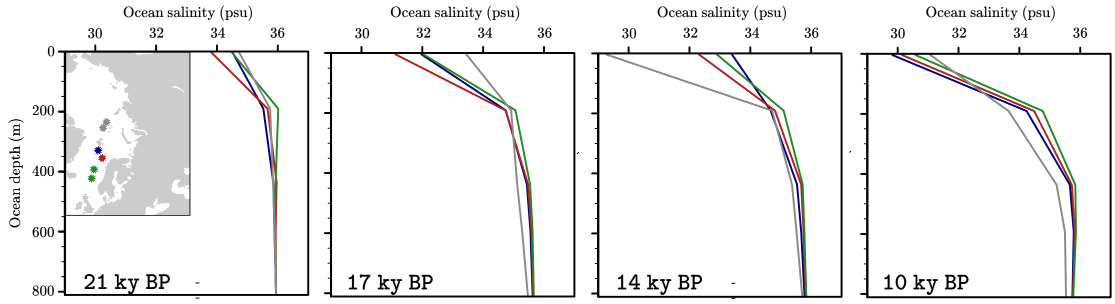

Figure 3: Ocean salinity vertical profiles derived from TraCE-21ka climate simulation Liu et al. (2009) at four different time slices (21, 17, 14, 10 ka). The vertical profiles are representative of the Norwegian Sea (green), South-Western Barents Sea (red), North-Western Barents Sea (blue) and Arctic Ocean (grey). In the inlay map the points selected in the TraCE-21ka grid to compute the vertical profiles are shown.

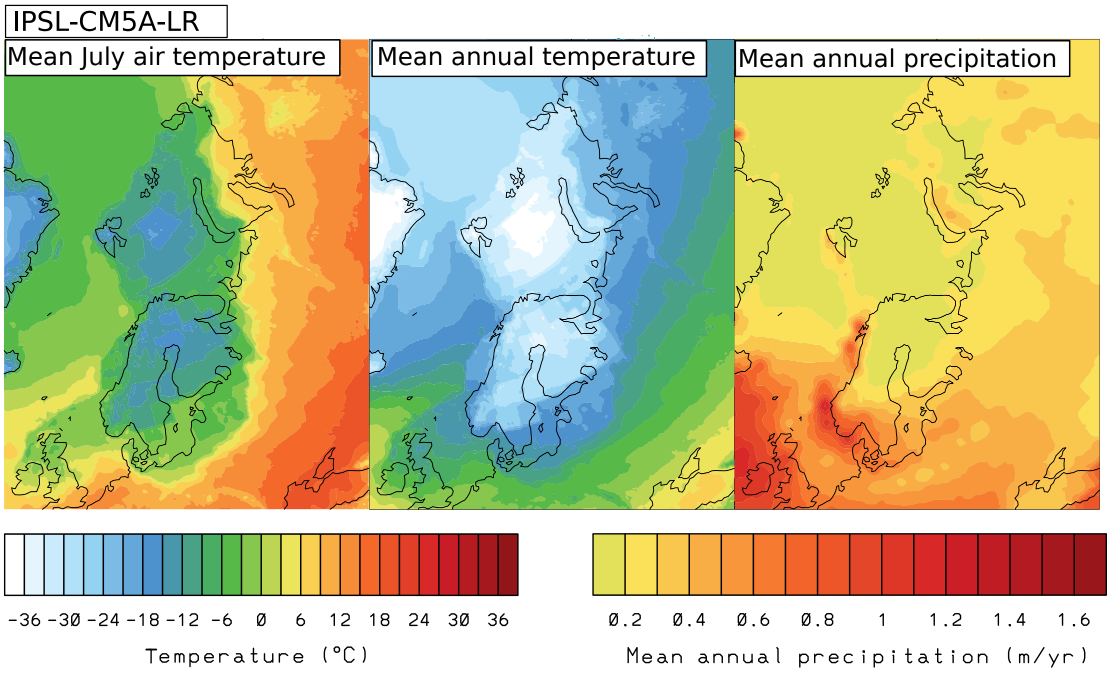

Figure 4: LGM climatology (July and annual mean near-surface air temperature and annual mean precipitation) simulated with the IPSL-CM5A-LR model Braconnot et al. (2012) used as initial climate snapshot in GRISLI and PSU simulations.

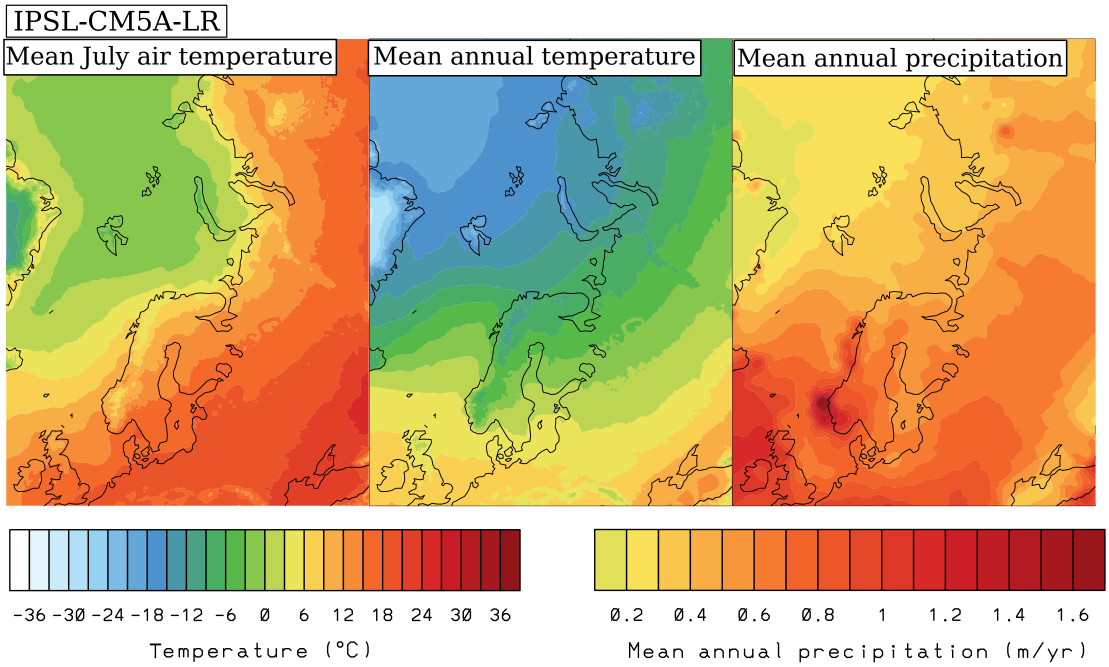

Figure 5: PI climatology (July and annual mean near-surface air temperature and annual mean precipitation) simulated with the IPSL-CM5A-LR model Braconnot et al. (2012) used as final climate snapshot in GRISLI and PSU simulations.

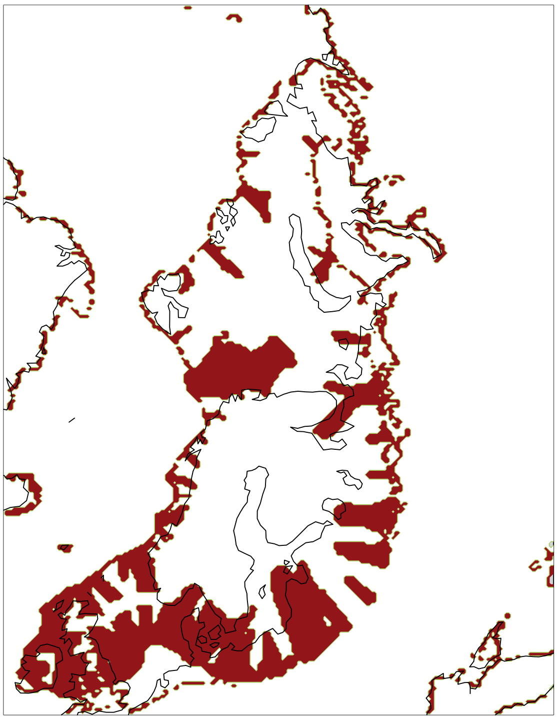

Figure 6: Ice stream area (red) simulated in GBvol simulation at the LGM.

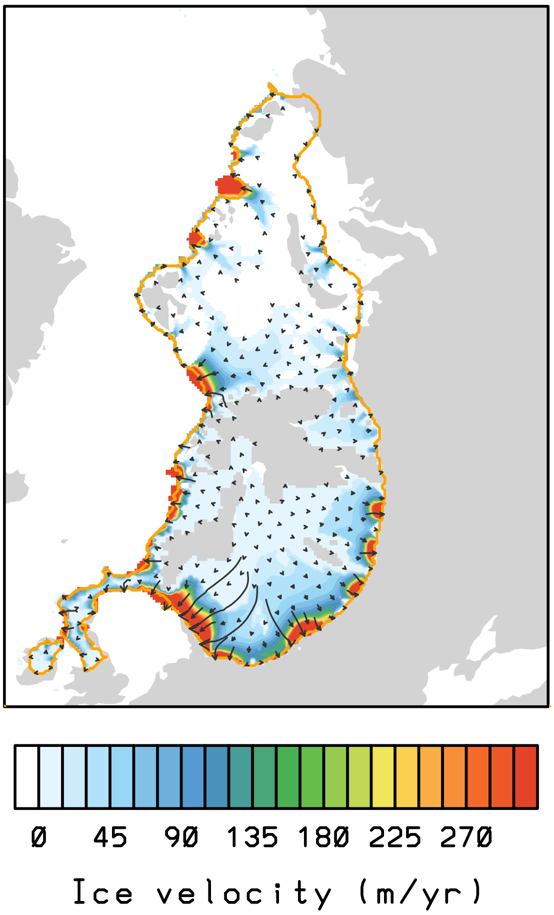

Figure 7: Ice velocity at the LGM in GBvol simulation.

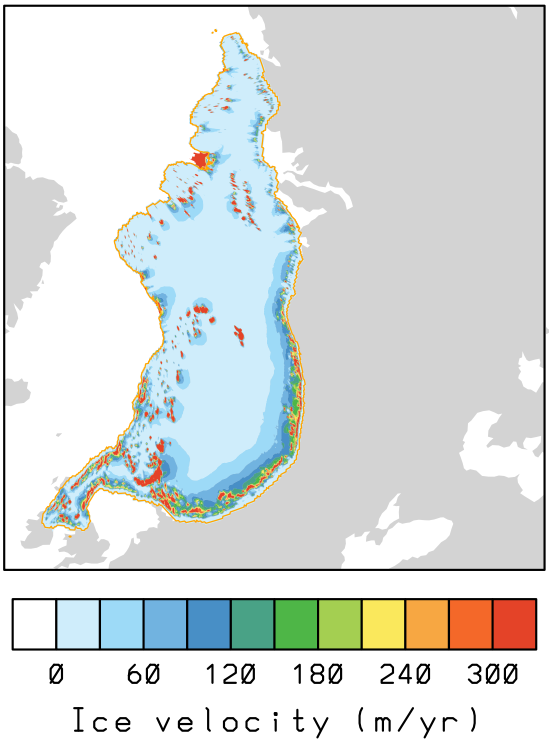

Figure 8: Ice velocity at the LGM in PBvol simulation.

Symbol	Description	Units	Value
ρ	Ice density.	kg/m^3	917
n	SIA Glen's flow law exponent.	-	3
n	SSA Glen's flow law exponent.	-	1
c	Ice heat capacity.	J/kg^{o}C	2009
κ	Ice thermal conductivity.	J/m^{o}Cyr	$2.1 \cdot 31557600$
ρ_w	Ocean water density.	kg/m^3	1025
$\overline{\lambda}$	Lapse-rate value used for downscaling.	^{o}C/km	0.005
$\overline{\gamma}$	Precipitation-correction factor used for downscaling.	1/^{o}C	0.05
ρ_{sw}	Subglacial water density.	kg/m^3	1000
K	Hydraulic conductivity.	m/s	10^{-6}
H_c	Thickness threshold for the calving criterion.	m	200
τ_f	Characteristic relaxation time of the astenosphere.	yr	3000

Table 1: List of the GRISLI parameter values common to all the simulations.

Symbol	Description	Units	Value
ρ	Ice density.	kg/m^3	917
c	Ice heat capacity.	J/kg^{o}C	2009
κ	Ice thermal conductivity.	J/m^{o}Cyr	$2.1 \cdot 31557600$
ρ_w	Ocean water density.	kg/m^3	1025
$\overline{\lambda}$	Lapse-rate value used for downscaling.	^{o}C/km	0.005
$\overline{\gamma}$	Precipitation-correction factor used for downscaling.	1/^{o}C	0.05
τ_f	Characteristic relaxation time of the astenosphere.	yr	3000
n	Glen's flow law exponent.	-	3
$\mathsf{E}_{\mathrm{SIA}}$	Shallow-ice approximation enhancement factor	-	10
$\mathsf{E}_{\mathrm{SSA}}$	Shallow-shelf approximation enhancement factor	-	3
c_f	"Frozen-bed" basal sliding coefficient.	m/yr Pa^2	10^{-20}
c_w	Full basal sliding coefficient.	m/yr Pa^2	$5 \cdot 10^{-8}$
H_c	Thickness threshold for the calving criterion.	m	200

Continued on the next page

Continued from previous page

Symbol	Description	Units	Value
cal_c	"Cold" calving rate.	m/yr	1
cal_w	"Warm" calving rate.	m/yr	80
λ	Lapse-rate value	$^{o}km^{-1}$	5.99
γ	Precipitation-correction factor	$^{o}C^{-1}$	0.43
F_m	Ocean basal melting parameter	-	$1.08 \cdot 10^{-3}$

Table 2: List of the PSU parameter values common to all the simulations.

**Parameters**	**Symbol**	**Units**	**Min.**	**Max.**	**GBvol**	**Pbvol**
Lapse-rate value	λ	$^{o}km^{-1}$	4.00	8.20	5.99	5.99
Precipitation-correction factor	γ	$^{o}C^{-1}$	0.03	0.10	0.43	0.43
SIA-enhancement factor	E_{SIA}	-	1.00	5.60	4.84	10.0
Basal drag coefficient	c_f	-	$1.0 \cdot 10^{-5}$	$10.0 \cdot 10^{-5}$	$7.1 \cdot 10^{-5}$	-
Ocean basal melting parameter	F_m	-	$0.02 \cdot 10^{-3}$	$1.56 \cdot 10^{-3}$	$1.08 \cdot 10^{-3}$	$1.08 \cdot 10^{-3}$

Table 3: Parameters included in the Latin Hypercube Sampling with GRISLI ISM. The minimum, maximum and the optimal values used in GBvol and GPvol simulations of each different parameter are shown.

Bibliography

Abe-Ouchi, A., Segawa, T., & Saito, F. (2007). Climatic conditions for modelling the northern hemisphere ice sheets throughout the ice age cycle. *Climate of the Past*, 3(3), 423–438.

Álvarez Solás, J., Montoya, M., Ritz, C., Ramstein, G., Charbit, S., Dumas, C., Nisancioglu, K., Dokken, T., & Ganopolski, A. (2011). Heinrich event 1: an example of dynamical ice-sheet reaction to oceanic changes. *Climate of the Past*, 7(4), 1297–1306.

Applegate, P. J., Parizek, B. R., Nicholas, R. E., Alley, R. B., & Keller, K. (2015). Increasing temperature forcing reduces the greenland ice sheet's response time scale. *Climate dynamics*, 45(7-8), 2001–2011.

Braconnot, P., Harrison, S. P., Kageyama, M., Bartlein, P. J., Masson-Delmotte, V., Abe-Ouchi, A., Otto-Bliesner, B., & Zhao, Y. (2012). Evaluation of climate models using palaeoclimatic data. *Nature Climate Change*, 2(6), 417–424.

Charbit, S., Ritz, C., & Ramstein, G. (2002). Simulations of northern hemisphere ice-sheet retreat:: sensitivity to physical mechanisms involved during the last deglaciation. *Quaternary Science Reviews*, 21(1), 243–265.

Colleoni, F., Quiquet, A., & Masina, S. (2016). *Long-term safety of a planned geological repository for spent nuclear fuel in Forsmark – Phase 2: Impact of ice sheet dynamics, climat forcing and multi-variate sensitivity analysis on maximum ice sheet thickness*. Technical Report SKB TR-14-21, Swedish Nuclear Fuel and Waste Management Co, Stockholm, Sweden.

Colleoni, F., Wekerle, C., & Masina, S. (2014). *Long-term safety of a planned geological repository for spent nuclear fuel in Forsmark – Phase 1: Estimate of maximum ice sheet thicknesses*. Technical Report SKB TR-14-21, Swedish Nuclear Fuel and Waste Management Co, Stockholm, Sweden.

Depoorter, M., Bamber, J., Griggs, J., Lenaerts, J., Ligtenberg, S., Van den Broeke, M., & Moholdt, G. (2013). Calving fluxes and basal melt rates of antarctic ice shelves. *Nature*, 502(7469), 89–92.

Dumas, C. (2002). *Modélisation de l'évolution de l'Antarctique depuis le dernier cycle glaciaire-interglaciaire jusqu'au futur: importance relative des différents processus physiques et rôle des données d'entrée*. PhD thesis, Université Joseph-Fourier-Grenoble I.

Fausto, R. S., Ahlstrøm, A. P., van As, D., & Steffen, K. (2011). Present-day temperature standard deviation parameterization for Greenland. *Journal of Glaciology*, 57(206), 1181–1183.

Hughes, A., Gyllencreutz, R., Lohne, O., Mangerud, J., & Svendsen, J. I. (2016). The last Eurasian ice sheets – a chronological database and time-slice reconstruction, DATED-1. *Boreas*, 45(1), 1–45.

Liu, Z., Otto-Bliesner, B. L., He, F., Brady, E. C., Tomas, R., Clark, P. U., Carlson, A. E., Lynch-Stieglitz, J., Curry, W., Brook, E., Erickson, D., Jacob, R., Kutzbach, J., & Cheng, J. (2009). Transient Simulation of Last Deglaciation with a New Mechanism for Bølling-Allerød Warming. *Science*, 325(5938), 310–314.

Ma, Y., Gagliardini, O., Ritz, C., Gillet-Chaulet, F., Durand, G., & Montagnat, M. (2010). Enhancement factors for grounded ice and ice shelves inferred from an anisotropic ice-flow model. *Journal of Glaciology*, 56(199), 805–812(8).

Marsiat, I. (1994). Simulation of the northern hemisphere continental ice sheets over the last glacial-interglacial cycle: experiments with a latitude-longitude vertically integrated ice sheet model coupled to zonally averaged climate model. *Paleoclimates*, 1, 59–98.

Martin, M., Winkelmann, R., Haseloff, M., Albrecht, T., Bueler, E., Khroulev, C., & Levermann, A. (2011). The potsdam parallel ice sheet model (pism-pik)–part 2: Dynamic equilibrium simulation of the antarctic ice sheet. *The Cryosphere*, 5(3), 727–740.

Peltier, W. (2004). Global glacial isostasy and the surface of the ice-age earth: the ICE-5G (VM2) model and GRACE. *Annual Review of Earth and Planetary Sciences*, 32(1), 111–149.

Peltier, W., Argus, D., & Drummond, R. (2015). Space geodesy constrains ice age terminal deglaciation: The global ice-6g_c (vm5a) model. *Journal of Geophysical Research: Solid Earth*, 120(1), 450–487.

Peyaud, V., Ritz, C., & Krinner, G. (2007). Modelling the early weichselian eurasian ice sheets: role of ice shelves and influence of ice-dammed lakes. *Climate of the Past Discussions*, 3(1), 221–247.

Pollard, D. & DeConto, R. M. (2012). Description of a hybrid ice sheet-shelf model, and application to Antarctica. *Geoscience Model Development*, 5, 1273–1295.

Purcell, A., Tregoning, P., & Dehecq, A. (2016). An assessment of the ice6g_c (vm5a) glacial isostatic adjustment model. *Journal of Geophysical Research: Solid Earth*, 121(5), 3939–3950.

Reeh, N. (1989). Parameterization of melt rate and surface temperature on the greenland ice sheet. *Polarforschung*, 59(3), 113–128.

Rignot, E., Jacobs, S., Mouginot, J., & Scheuchl, B. (2013). Ice-shelf melting around antarctica. *Science*, 341(6143), 266–270.

Stokes, C. R. & Tarasov, L. (2010). Ice streaming in the laurentide ice sheet: A first comparison between data-calibrated numerical model output and geological evidence. *Geophysical Research Letters*, 37(1).

Stone, E., Lunt, D., Rutt, I., & Hanna, E. (2010). Investigating the sensitivity of numerical model simulations of the modern state of the greenland ice-sheet and its future response to climate change. *The Cryosphere*, 4(3), 397.

Tarasov, L. & Peltier, W. R. (2002). Greenland glacial history and local geodynamic consequences. *Geophysical Journal International*, 150(1), 198–229.

Wekerle, C., Colleoni, F., Näslund, J.-O., Brandefelt, J., & Masina, S. (2016). Numerical reconstructions of the penultimate glacial maximum northern hemisphere ice sheets: sensitivity to climate forcing and model parameters. *Journal of Glaciology*, 62(234), 607–622.
